# Asthma and increased carotid intima-media thickness: A systematic review and meta-analysis

**DOI:** 10.17305/bb.2026.14164

**Published:** 2026-04-24

**Authors:** Mohammed Haroun, Mohamed Ellebedy, Abduraheem F A Farah, Waleed K K Khalid, Mazin M A Abdelmutalab, Omer H T Mohmmed, Aymen Abdalla, Sagad O O Mohamed

**Affiliations:** 1 Internal Medicine, Ascension Saint Joseph Hospital, Chicago, IL, USA; 2 Faculty of Medicine, Sohag University, Sohag, Egypt; 3 Department of Anatomical Sciences, St George’s University, West Indies, Grenada; 4 Department of Emergency Medicine, Khoula Hospital, Muscat, Oman; 5 Hamad Medical Corporation, Doha, Qatar; 6 Faculty of Medicine, University of Gezira, Wad Madani, Sudan; 7 Faculty of Medicine, University of Khartoum, Khartoum, Sudan

**Keywords:** Asthma, carotid intima-media thickness, CIMT, cardiovascular risk, systematic review, meta-analysis

## Abstract

Asthma has been associated with early vascular changes that elevate the risk of atherosclerosis and cardiovascular diseases. Carotid intima-media thickness (CIMT) serves as a non-invasive marker for subclinical atherosclerosis and cardiovascular risk. However, existing literature presents conflicting results regarding CIMT measurements in asthmatic patients. This study aims to evaluate whether CIMT is elevated in asthma patients compared to healthy controls and to explore potential modifiers. We conducted a systematic review following PRISMA guidelines, performing a literature search across PubMed, Web of Science, ScienceDirect, and the WHO VHL. We calculated pooled standardized mean differences (SMD) with 95% confidence intervals (CI) to assess the differences in CIMT values. Heterogeneity was evaluated using the I^2^ statistic, and subgroup analyses and meta-regression were utilized to identify sources of heterogeneity. Our review included a total of 10 studies. The analysis indicated significantly higher CIMT values in asthma patients compared to healthy controls, with a pooled SMD of 0.65 (95% CI: 0.19 to 1.12, *P ═* 0.005). A limited number of studies addressed various factors, such as disease severity and the use of inhaled corticosteroids, which may influence CIMT measurements. Notably, asthmatic patients, particularly children and adolescents, exhibited higher CIMT values compared to healthy controls, suggesting a potential association with subclinical atherosclerosis in this demographic. However, these findings should be interpreted with caution due to the observational nature of the included studies and the risk of residual confounding. Further longitudinal research is necessary to elucidate the effects of disease characteristics and treatment on vascular health.

## Introduction

Both respiratory and cardiovascular diseases (CVD) represent significant global health challenges, contributing to a considerable burden worldwide [1, 2]. Asthma, a chronic respiratory condition, affects approximately 300 million individuals globally [2], while atherosclerotic CVD remains the leading cause of mortality, accounting for around 17.9 million deaths annually [1]. The pathogenesis of asthma is complex and multifactorial, characterized by persistent inflammation, oxidative stress, and immune dysregulation [3]. These mechanisms may also lead to vascular alterations and heightened cardiovascular risk [3, 4]. Both asthma and CVD feature a chronic inflammatory response, marked by elevated levels of systemic inflammatory markers such as C-reactive protein, interleukin-6, and tumor necrosis factor-alpha. These markers contribute to airway inflammation in asthma and endothelial dysfunction in early atherosclerosis [5].

Research has explored the association between asthma and CVD, indicating that asthma may serve as a risk factor for atherosclerosis [6]. However, early detection of subclinical atherosclerosis remains challenging. Various imaging modalities and biochemical methods have been employed to assess this condition [7]. Among these, carotid intima-media thickness (CIMT) is a non-invasive marker utilized to evaluate early vascular remodeling and predict future cardiovascular events [8]. CIMT, defined as the distance between the lumen–intima interface and the media–adventitia interface of the arterial wall, serves as an ultrasound marker for assessing the severity and progression of atherosclerosis, providing a preclinical measure of vascular health, particularly in individuals with chronic conditions such as diabetes, hypertension, and hyperlipidemia [9, 10].

Numerous observational studies have examined the relationship between asthma and CIMT, revealing that asthmatic patients tend to exhibit higher CIMT values compared to non-asthmatic individuals, suggesting an increased risk of atherosclerosis and CVD. Some studies report significantly elevated CIMT in asthmatic patients, while others indicate minimal differences compared to healthy individuals. Additionally, the relationship between CIMT and key disease-related factors, such as disease severity, remains uncertain. To address these discrepancies, a systematic review of existing research could elucidate conflicting findings and identify existing knowledge gaps.

This systematic review and meta-analysis primarily aim to evaluate differences in CIMT between patients with asthma and healthy controls. Secondary objectives include a narrative synthesis of factors associated with CIMT, including disease severity, duration, and treatment.

## Materials and methods

### Search approach and study inclusion criteria

This systematic review was conducted according to the guidelines set forth by the Preferred Reporting Items for Systematic Reviews and Meta-Analyses (PRISMA) statement [11]. The review protocol was prospectively recorded on the Open Science Framework platform (https://osf.io/yvqkn). To identify all relevant published research, we performed an extensive electronic literature search across PubMed, Web of Science, ScienceDirect, and the World Health Organization Virtual Health Library Regional Portal (WHO VHL), without imposing restrictions based on geographical location or publication date, to encompass a broad spectrum of pertinent studies.

The search included published articles from each database’s inception until March 2025, utilizing the following keywords across all fields: (“Asthma”) AND (“carotid intima media thickness” OR “carotid intima-media thickness” OR “IMT” OR “CIMT” OR “C-IMT” OR “CAIMT” OR “Intima-media complex”). The complete search strategy, inclusive of Boolean operators and field tags, is detailed in Supplementary File 1. Additionally, we reviewed the reference lists of all included articles to identify any additional relevant studies that may have been overlooked during the primary search. Alongside database searches, we utilized Google Scholar to capture potential grey literature pertinent to our research question. All identified publications were uploaded to EndNote software for initial screening of titles and abstracts, as well as for the removal of duplicates.

### Inclusion and exclusion criteria

Following the literature search, the selection process employed a two-step approach. Initially, three independent reviewers screened the titles and abstracts of all identified articles to select potentially relevant studies. Publications that passed this initial screening underwent a full-text assessment to determine compliance with the predetermined inclusion criteria. Specifically, we included observational studies (cross-sectional, case-control, and cohort studies) that provided data on one or more of the following outcomes: I) CIMT measurements in individuals with asthma compared to healthy counterparts, II) associations between CIMT measurements in asthmatic patients and identified biomarkers, and III) associations between CIMT values and asthma severity. Conversely, studies were excluded if they met any of the following criteria: lack of relevant data, case reports, editorials, reviews, conference abstracts, or publications in languages other than English.

### Quality assessment and data extraction

The methodological quality and potential bias of the included studies were evaluated using the standardized Joanna Briggs Institute critical appraisal checklists (https://jbi.global/critical-appraisal-tools). This validated assessment tool facilitates systematic evaluation of potential biases across various domains, including study design, implementation methods, and analytical approaches. Data extracted from each study included authors, year, region, number of patients, age group of participants, mean age, and CIMT measurements. In instances where a study reported median and interquartile range, mean and standard deviation (SD) were estimated using a previously established method by Wan et al. [12]. When a study presented multiple means and SD across different patient subgroups, we combined these to generate a single set of values for each study [13]. Any discrepancies encountered during the quality assessment and data extraction process were resolved through discussion.

### Statistical analysis

Statistical analyses were conducted using R version 4.4.3 to calculate the pooled standardized mean difference (SMD) and its 95% confidence intervals (CI). Since CIMT was reported in varying units and measurement protocols across studies, the SMD was deemed the appropriate standardized metric for pooling. A random-effects model employing the restricted maximum likelihood (REML) estimation method was utilized to calculate the pooled effect size, accounting for anticipated heterogeneity among studies, evaluated using the I^2^ test. An additional sensitivity analysis using the Hartung–Knapp adjustment was performed to provide more conservative CIs. To assess for publication bias, we employed both the Begg and Mazumdar rank correlation test and Egger’s regression test, along with a visual examination of funnel plots to identify asymmetries that could indicate publication bias [14–16]. Leave-one-out sensitivity analyses were conducted to evaluate the robustness of the meta-analyses by assessing the influence of individual studies on the outcomes. Additionally, meta-regression subgroup analyses were performed to examine how specific study characteristics correlate with variations in effect sizes. The significance level for all analyses was set at 0.05.

## Results

### Studies characteristics

The schematic flow of the study identification and selection process is illustrated in Figure 1. The initial electronic database search yielded a total of 265 records. After removing duplicates, 181 studies remained. These studies underwent a comprehensive title and abstract screening process, resulting in the exclusion of 170 studies due to irrelevance. The full texts of the remaining records were subsequently screened, culminating in the selection of 10 studies for inclusion in the systematic review and meta-analysis [17–26].

**Figure 1.**
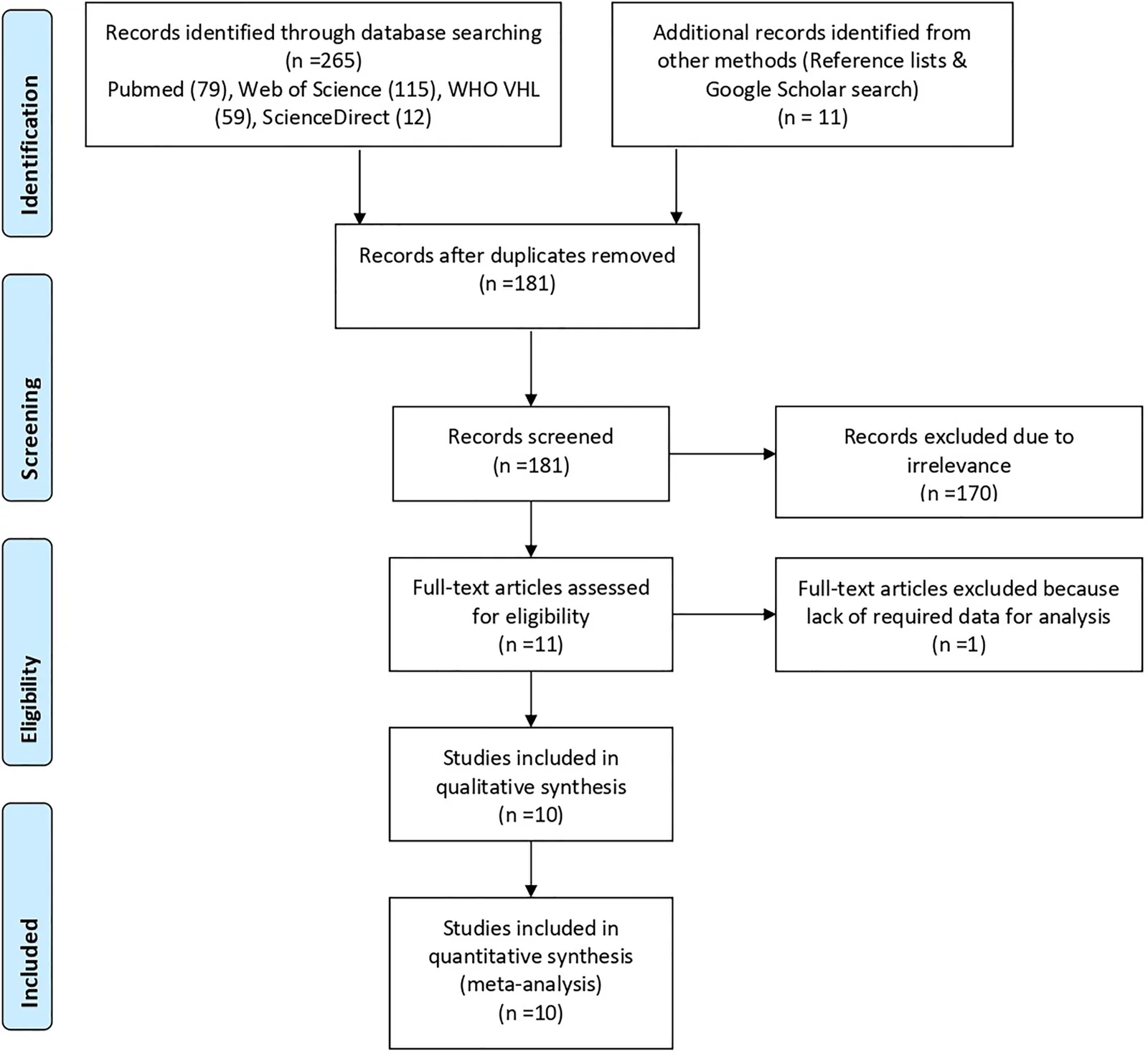
**Flowchart illustrating the study selection process**.

Study publications spanned from 2009 to 2022 and included patients from seven countries, comprising a total of 2,976 participants, of which 774 were asthmatic patients. Among the included studies, six focused on pediatric and adolescent populations with asthma, while four involved adult populations. CIMT measurements were performed using high-resolution ultrasound with varying protocols (Table 1). The primary characteristics of the selected studies, along with risk of bias assessments, are presented in Tables 1 and 2.

**Table 1:** Patient characteristics and carotid intima-media thickness measurement methods

**Study**	**Patient characteristics**	**CIMT measurement**	**Asthma severity and ICS use**	**Adjusted confounders**
Cakmack et al.	Children aged 6–15 years attending pediatric allergy unit for at least one year.	Bilateral CCA; single blinded examiner; high-resolution Doppler ultrasound (Logiq 7 Pro, 12-MHz probe); mean of measurements 1 cm proximal and distal to maximum thickness near carotid bifurcation.	Mild intermittent. ICS use was not reported	Age, sex and BMI
Dratva et al.	Adolescents from a population-based Swiss cohort.	Bilateral CCA; automatic contour detection program; 1-cm segment proximal to bulb; end-diastolic average CIMT across several heart cycles.	Not reported	Age, sex, puberty birth/pregnancy outcomes, early childhood inflammatory exposures, and parental factors
Karakaya et al.	Children ≥6 years with mild persistent asthma; not on steroids or in acute exacerbation.	Right CCA; single blinded examiner; Samsung H60 system, 4–9 MHz linear probe; posterior wall, 1.5 cm proximal to bifurcation; mean of three heart cycles.	Mild persistent. ICS use was not reported	Age, sex, weight, height, and BMI
Olmez et al.	Children aged 8–18 years with mild or moderate asthma.	Far wall of left CCA; 1–2 cm segment proximal to bulb; mean of three distinct measurements.	Mild–moderate. ICS use was not reported	Age, sex, weight, height, and weight-for-height index
Otsuki et al.	Adults (<65 years) on regular ICS use for ≥2 years; no oral or intravenous steroids.	Bilateral CCA; single blinded operator; high-resolution B-mode ultrasound (LOGIQ 200 MD, GE); three sites: greatest thickness, 1 cm upstream, 1 cm downstream; mean of all six measurements.	Not reported. All patients on regular ICS	Age, sex, diabetes, hypertension, dyslipidemia, and smoking. Stepwise regression: BMI, daily ICS dose, asthma duration, and lung function
Pacholczak-Madej et al.	Stable adults with asthma; no exacerbation in preceding 6 months.	CCA; two independent blinded experts; Siemens Acuson Sequoia 512, 10-MHz probe; mean of three consecutive measurements per operator.	Mild–moderate (clinically stable)	Age, sex, BMI, and comorbidities (HTN, DM, hypercholesterolemia).
Podgorski et al.	Adults >30 years on regular ICS; mostly well-controlled, moderate severity.	Bilateral CCA to bulb; far wall; semi-automated border-detection (EchoPac PC); mean IMT over three cardiac cycles.	Moderate, well-controlled. Patients on ICS.	Age, weight, and systolic blood pressure
Tattersall et al.	Children from the COAST birth cohort at increased risk for asthma and atopy; high rates of medication use.	Right CCA; B-mode ultrasound (Siemens Acuson S2000, 9L4 probe); far wall; distal 1 cm of right CCA and proximal 1 cm of carotid bifurcation; semi-automated maximum segmental IMT from end-diastolic frames.	Not reported. Patients on ICS	Age, gender, race, systolic blood pressure, and BMI
Winder et al.	Children aged 14–16 years; mostly allergic asthma.	Bilateral CCA; far wall; distal 4 cm; high-resolution ultrasound (GE 12L-RS probe, 6–13 MHz, Vivid q); single blinded rater; three measurements per side on stored images; mean of six measurements.	Not reported (mostly allergic)	Age, sex, size, waist circumference, systolic BP, physical activity, smoking, LDL-C, ALT, and CRP
Yilmaz et al.	Adults admitted to pulmonary medicine department; 25.3% had severe asthma; median asthma duration 12 months.	Bilateral CCA; far wall; 1 cm proximal to bifurcation; B-mode duplex ultrasound (Philips EPIQ 7, L12-3 probe); two blinded operators; three adjacent sites 1 mm apart; mean of all measurements.	Mild–severe (25.3% severe). Patients on ICS.	Age, sex, and BMI

**Abbreviations:** ALT, alanine aminotransferase; BMI, body mass index; BP, blood pressure; CCA, common carotid artery; CIMT, carotid intima-media thickness; COAST, Childhood Origins of Asthma; CRP, C-reactive protein; DM, diabetes mellitus; HTN, hypertension; ICS, inhaled corticosteroids; IMT, intima-media thickness; LDL-C, low-density lipoprotein cholesterol.

**Table 2:** Baseline characteristics of studies included in the review

**Study**	**Year**	**Country**	**Sample size**	**Male** **%**	**Age mean**	**Associated factors with CIMT**	**Quality assessment**
Cakmack et al.	2010	Turkey	278	90.9	9.05	Asthma diagnosis and markers of oxidative stress (oxidative stress status and total peroxide concentrations of the plasma)	7/10
Dratva et al.	2018	Switzerland	257	47	15	Asthma diagnosis, gender (male), and predicted FEV1%	9/11
Karakaya et al.	2020	Turkey	49	48.5	9.1	Asthma diagnosis and duration of asthma	6/10
Olmez et al.	2009	Turkey	56	54.5	12.88	Asthma diagnosis but not asthma severity.	8/10
Otsuki et al.	2010	Japan	300	50.67	55.2	CIMT was significantly lower in asthmatics patients treated with inhaled corticosteroid	10/10
Pacholczak-Madej et al.	2021	Poland	154	28.26	54	Asthma diagnosis, asthma severity, and Inflammatory biomarkers such as interleukin-6 and 10	8/10
Podgorski et al.	2017	Poland	204	31.4	56.9	Predicted FEV1%. Reduced CIMT in asthmatic patients treated with inhaled corticosteroid but the association was not statistically significant.	8/10
Tattersall et al.	2018	USA	89	58	15.3	Asthma diagnosis and atopic disease presence	9/11
Winder et al.	2022	Austria and Italy	1506	43.5	17.8	Allergic asthma	10/11
Yilmaz et al.	2017	Turkey	83	46.1	44.29	Asthma severity, duration of symptoms, and predicted FEV1%	8/10

**Abbreviations:** CIMT, carotid intima-media thickness; FEV1, forced expiratory volume in 1 second.

### CIMT measurements among patients with asthma

We conducted a meta-analysis to evaluate the differences in mean CIMT levels between patients with asthma and healthy individuals. The results indicated that individuals with asthma exhibited significantly higher CIMT measurements compared to healthy counterparts, with a pooled SMD of 0.65 (95% CI: 0.19–1.12, *P ═*.005) (Figure 2). The I^2^ test demonstrated a high level of heterogeneity (91.6%). The prediction interval was wide (−1.05 to 2.35), reflecting considerable between-study heterogeneity. Sensitivity analysis employing the Hartung–Knapp adjustment yielded consistent results, with a pooled SMD of 0.65 (95% CI: 0.10–1.20, *P ═* 0.024), confirming the robustness of the findings despite wider CIs. The publication bias assessment yielded significant results for Egger’s test (*P ═* .022) but not for the Begg’s test (*P ═* .128). However, no definitive conclusions regarding the presence or absence of publication bias can be drawn due to the limited number of studies (Figure 3).

**Figure 2.**
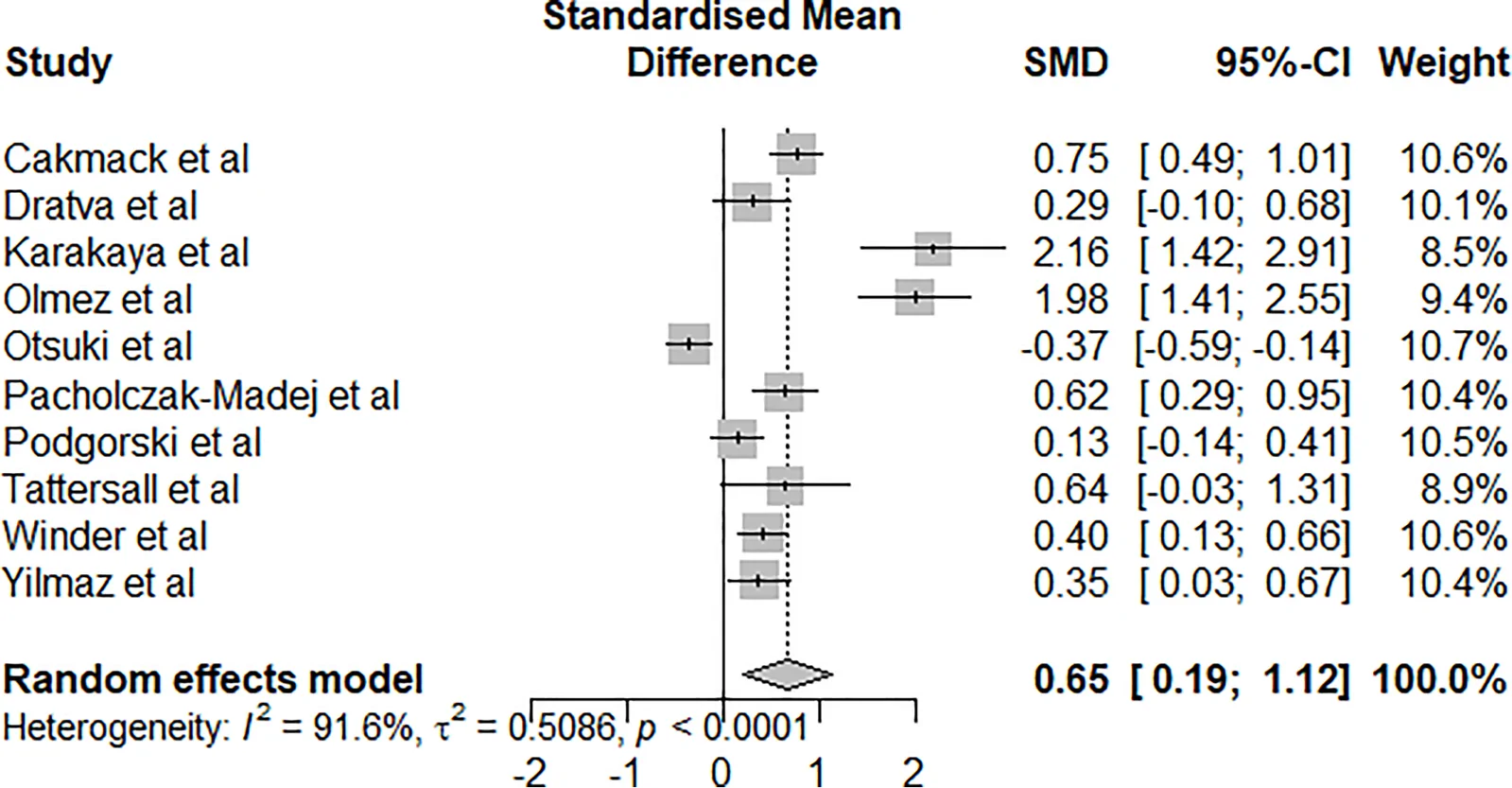
**Forest plot of the pooled standardized mean difference in carotid intima-media thickness between patients with asthma and healthy controls.** Individual study estimates are presented as squares proportional to study weight, with horizontal lines indicating 95% CIs. The diamond represents the pooled random-effects estimate and shows significantly higher carotid intima-media thickness in patients with asthma than in healthy controls (SMD = 0.65, 95% CI: 0.19–1.12). Positive SMD values indicate higher carotid intima-media thickness in the asthma group, whereas negative values indicate lower values relative to controls. Substantial between-study heterogeneity was observed (I^2^ ═ 91.6%, τ^2^ ═ 0.5086, *P <* 0.0001). **Abbreviations:** CI, confidence interval; CIMT, carotid intima-media thickness; SMD, standardized mean difference.

**Figure 3.**
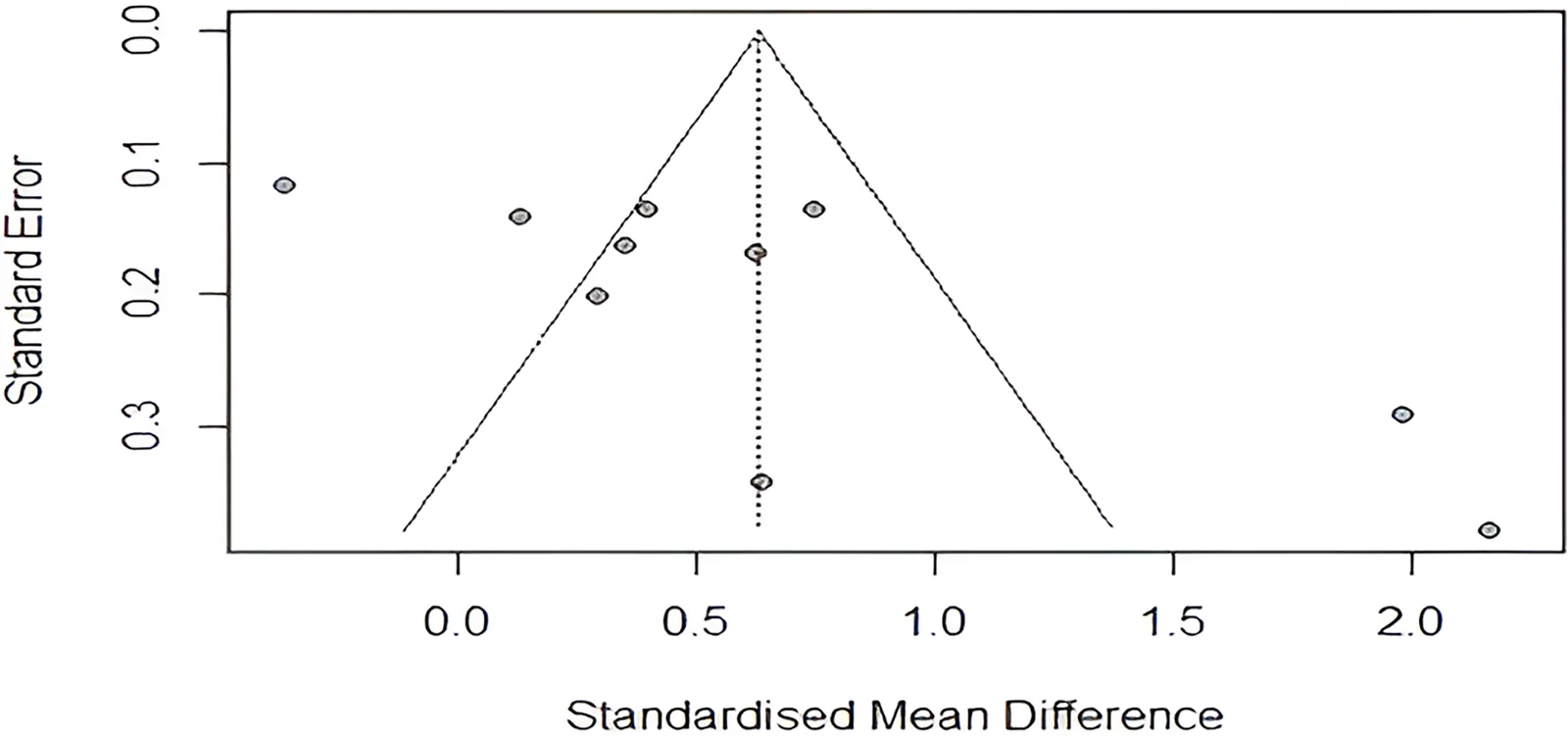
**Funnel plot assessing potential publication bias in the meta-analysis of carotid intima-media thickness in patients with asthma versus healthy controls.** Each circle represents an individual study plotted according to its standardized mean difference and standard error. The vertical dotted line indicates the pooled effect estimate, while the diagonal dashed lines represent the 95% pseudo-confidence limits around the summary effect. Visual inspection suggests some asymmetry, particularly among smaller studies with larger effect sizes; however, in line with the statistical results reported in the text, this finding should be interpreted cautiously given the limited number of included studies. **Abbreviations:** CIMT, carotid intima-media thickness; SMD, standardized mean difference.

### Moderators of heterogeneity

Meta-regression and subgroup analyses were performed to determine whether specific variables, including the number of patients, sex, publication year, and age groups, influenced heterogeneity in the meta-analysis. Results indicated that the number of patients had a negative moderating effect on the outcome (coefficient ═ –0.011, *P ═* 0.008). However, this association was weak and should be interpreted as exploratory. Conversely, the male ratio (coefficient ═ –0.001, *P ═* 0.358) and publication year (coefficient ═ –0.004, *P ═* 0.915) exhibited no significant moderating effect on the outcome. Subgroup analysis based on age groups revealed a significant difference in the pooled effect size (*P ═* 0.018), with a higher effect size observed in pediatric asthma. The highest pooled SMD was noted in studies involving asthmatic children (SMD = 0.975), while the analysis of four adult-based studies showed a lower difference between asthmatic patients and non-asthmatic participants (SMD = 0.175).

Additionally, a leave-one-out sensitivity analysis was conducted to evaluate the influence of individual studies on the pooled effect estimate. All re-analyses, where each study was removed one at a time, yielded a statistically significant pooled effect estimate, indicating consistency in the analysis. The effect size from each individual study did not cause excessive variation in the pooled estimate. Notably, the removal of the studies by Karakaya et al. and Olmez et al. resulted in significantly lower, yet still significant, estimates (SMD = 0.495, *P ═* 0.006 and SMD = 0.484, *P ═* 0.005, respectively). Conversely, removing the study by Otuski et al. led to a higher, but still significant, estimate (SMD = 0.749, *P <* 0.001). To assess the potential impact of approximation error introduced by median-to-mean conversion, a sensitivity analysis excluding the two studies requiring this conversion (Karakaya et al. and Winder et al.) was performed. The pooled estimate remained statistically significant following their exclusion, albeit with a reduced effect size (SMD = 0.55, 95% CI: 0.069–0.975, *P ═* 0.024).

### Associated factors with CIMT levels in asthmatic patients

Several studies evaluated the relationship between CIMT and various factors, including asthma severity, duration, treatment, and predicted forced expiratory volume in 1 second (FEV1%). These investigations revealed discrepancies in how these factors influenced CIMT measurements among asthmatic patients (Table 2). Karakaya et al. identified a significant positive correlation between symptom duration and CIMT in children with mild persistent asthma, suggesting that longer symptom duration correlates with increased CIMT. Yilmaz et al. reported similar significant differences in CIMT measurements among groups of mild, moderate, and severe asthmatic patients. In contrast, Podgorski et al. found no significant differences in adult patients. The impact of inhaled corticosteroids was evaluated in two studies, which presented conflicting results regarding their effects on CIMT measurements. Otsuki et al. provided the most compelling evidence, indicating a protective role for inhaled corticosteroids, potentially in a dose-dependent manner, resulting in lower CIMT compared to untreated individuals. However, Podgórski et al. found no significant differences after adjustment (Table 2).

## Discussion

The findings suggest that asthmatic patients exhibit higher CIMT measurements than non-asthmatic individuals, indicating a potential need for further investigation into cardiovascular risk associated with asthma. These results imply that asthmatic patients may have an increased risk of developing early-onset atherosclerosis and, ultimately, CVD. CIMT serves as a surrogate marker and a strong predictor of CVD [27–29]; a 0.10 mm increase in CIMT is associated with an 18% and 15% increased risk of developing stroke and myocardial infarction, respectively [27–29]. While this meta-analysis indicates that asthma is linked to higher CIMT compared to healthy controls, the magnitude of this association varies significantly across studies, as evidenced by the high heterogeneity. Thus, the pooled estimate should be interpreted cautiously and regarded as an average effect across diverse populations and study designs rather than a consistent effect size.

The observed high degree of heterogeneity likely reflects variances in study populations, including age groups, asthma phenotypes, comorbidities, and treatment practices, as well as differences in CIMT measurement protocols. These factors may influence baseline CIMT values and the strength of the observed association, limiting the generalizability of the pooled estimate.

The findings are consistent with various studies that have examined the association between asthma and coronary heart disease and CVD risk [5, 30]. A population-based study also indicated that asthma is a risk factor for myocardial infarction [31]. This association may be attributed to circulating inflammatory markers in asthmatic patients, which can lead to endothelial damage and a predisposition to atherosclerosis [32, 33]. Our subgroup analysis and the results from included studies demonstrated that the association between asthma and increased CIMT is particularly pronounced in pediatric patients. Chronic inflammation during childhood, potentially exacerbated by persistent asthma, may accelerate vascular changes at a critical developmental stage. Consequently, children exposed to atherosclerotic risk factors may exhibit advanced vascular aging, as reported by Le et al. [34]. Furthermore, the association between asthma and CIMT may be more readily detectable in children, as it is less likely to be obscured by factors that influence CIMT in adults, such as the use of antihypertensive and lipid-lowering medications. Nonetheless, it is important to acknowledge that this finding may be limited by the small number of studies.

A substantial degree of heterogeneity was observed in the subgroup analysis. While meta-regression and subgroup analyses were conducted to explore potential sources of heterogeneity, these findings should be considered exploratory due to the limited number of included studies. A significant source of heterogeneity in this meta-analysis stems from the lack of standardization in CIMT measurement protocols. Included studies varied in terms of assessed arterial segments, laterality (unilateral vs. bilateral), number of measurements, operator dependence, and analytical software. These methodological discrepancies can influence absolute CIMT values and may significantly contribute to the variability in effect sizes observed across studies. Other factors, such as differences in participants’ baseline characteristics, including asthma severity, comorbidities, and corticosteroid use, may also explain this heterogeneity. Patients with persistent asthma tend to exhibit higher levels of systemic inflammatory markers compared to those with intermittent asthma or without asthma [33].

An overlap may exist between the inflammatory pathogenesis of asthma and atherosclerosis through the 5-lipoxygenase pathway. Asthmatic inflammation is partially mediated by this pathway, which catalyzes the conversion of arachidonic acid into leukotrienes—pro-inflammatory substances secreted by immune cells that contribute to both acute and chronic inflammation [35]. In the bronchioles, leukotrienes facilitate inflammation by constricting smooth muscle, inducing tissue edema, and signaling eosinophil migration [36]. In atherosclerosis, atheromatous plaques express 5-lipoxygenase, with plaque instability correlating positively to elevated levels of 5-lipoxygenase and leukotrienes [35, 37]. Moreover, inhibition of 5-lipoxygenase has demonstrated a protective effect against myocardial infarction and atherosclerosis in animal models [38, 39]. Consequently, patients with persistent asthma may exhibit higher CIMT measurements and an increased risk of developing atherosclerosis and CVD compared to individuals with intermittent asthma or those without asthma. However, further research is necessary to elucidate the relationship between CIMT and asthma severity and duration.

Regarding comorbid conditions, prior studies have indicated that CIMT is associated with hypertension and obesity. A nationwide study conducted in Germany found a significant association between both conditions and increased CIMT [40]. Thus, additional research is warranted to explore the connection between CIMT and asthmatic patients with comorbidities such as hypertension and obesity.

Corticosteroids remain a cornerstone in the management of bronchial asthma, with inhaled corticosteroids being the first-line treatment for most patients [41]. In contrast to oral corticosteroids, inhaled corticosteroids at appropriate doses exhibit minimal systemic side effects [42]. The impact of inhaled corticosteroids on CVD progression has shown inconsistency, with limited studies indicating potential benefits on atherosclerotic progression in asthmatic patients [17]. Therefore, further investigation is required to assess the effects of inhaled corticosteroids on CIMT and the progression of atherosclerosis.

To our knowledge, this is the first meta-analysis examining the relationship between asthma and CIMT. The results remained significant even after conducting a leave-one-out sensitivity analysis. However, several limitations warrant consideration. First, the studies included in the meta-analysis exhibited high heterogeneity, likely due to variations in study populations, asthma severity and duration, and CIMT measurement protocols, which may restrict the generalizability of the findings. It is important to acknowledge that combining two biologically distinct age groups into a single pooled estimate represents a methodological limitation. The notably lower effect size in adults compared to children suggests that the overall pooled SMD is heavily influenced by pediatric data and may not accurately reflect the association within adult asthmatic populations. Second, most of the included studies are cross-sectional in nature, which limits the ability to establish causal relationships. Furthermore, not all studies adjusted for potential confounders such as obesity, hypertension, smoking, and dyslipidemia, which also impact CIMT. Unmeasured confounders may partially contribute to the observed results. The wide prediction interval further limits the generalizability of these findings. This variability is expected due to clinical differences across studies, including variations in asthma severity and treatment exposure. Therefore, more rigorously designed studies are essential. Lastly, the relatively small number of studies in the subgroup analysis may affect its statistical power.

## Conclusion

Asthmatic patients, particularly children and adolescents, demonstrate higher CIMT measurements compared to healthy controls, indicating a potential increased risk of subclinical atherosclerosis in this demographic. Nonetheless, this finding should be interpreted cautiously due to the observational nature of the included studies and the possibility of residual confounding. Consequently, larger-scale studies are necessary to further investigate the relationship between CIMT, asthma, and related factors.

## Supplemental data

**Search strategy**

**1. PubMed (MEDLINE)**

 (“asthma[tw] OR “bronchial asthma”[tw])

 AND

 (“carotid intima media thickness”[tw]

 OR “carotid intima-media thickness”[tw]

 OR CIMT [tw] 

 OR “C-IMT”[tw] 

 OR IMT [tw]

 OR “intima media thickness”[tw]

 OR “intima-media complex”[tw])

**2. Web of Science**

 TS= (asthma OR “bronchial asthma”)

 AND

 TS= (“carotid intima media thickness”

 OR “carotid intima-media thickness”

 OR CIMT

 OR “C-IMT”

 OR IMT 

OR “intima media thickness” 

 OR “intima-media complex”)

**3. Science Direct**

 TITLE-ABSTR-KEY (asthma OR “bronchial asthma”)

 AND

 TITLE-ABSTR-KEY (“carotid intima media thickness” 

 OR “carotid intima-media thickness” 

 OR CIMT 

 OR “C-IMT” 

 OR IMT 

 OR “intima media thickness” 

 OR “intima-media complex”) 

**4. WHO Global Health Library (VHL)**

 TITLE-ABSTRACT (asthma OR “bronchial asthma”)

 AND

 TITLE-ABSTRACT (“carotid intima media thickness” 

 OR “carotid intima-media thickness” 

 OR CIMT 

 OR “C-IMT” 

 OR IMT 

 OR “intima media thickness” 

 OR “intima-media complex”) 

**Table S1:** Complete study-level data

**Study**	**Asthma group, *n***	**Mean**	**SD**	**Control group, *n***	**Mean**	**SD**	**Required median-mean conversion**
Cakmack et al.	84	0.46	0.06	194	0.42	0.05	No
Dratva et al.	28	0.527	0.063	229	0.513	0.046	No
Karakaya et al.	33	1.53	0.54	16	0.53	0.16	Yes
Olmez et al.	56	0.45	0.05	24	0.35	0.05	No
Pacholczak-Madej et al.	92	0.73	0.11	62	0.65	0.15	No
Podgorski et al.	102	0.65	0.15	102	0.63	0.15	No
Tattersall et al.	34	597	65	12	558	42	No
Winder et al.	58	431.4	48.81	1158	412.23	48.25	Yes
Yilmaz et al.	83	5.52	0.44	71	5.36	0.47	No

**Abbreviation:** SD, standard deviation.

## Data Availability

Dataset used for this study is available from the corresponding author upon reasonable request.
